# Acutely Onset Amiodarone-Induced Angioedema in a Patient with New Atrial Fibrillation

**DOI:** 10.1155/2014/321587

**Published:** 2014-12-25

**Authors:** Hossein Vakili, Isa Khaheshi, Mehdi Memaryan, Habib Haybar, Shooka Esmaeeli

**Affiliations:** ^1^Cardiovascular Research Center, Modarres Hospital, Shahid Beheshti University of Medical Sciences, Tehran, Iran; ^2^Cardiovascular Research Center, Ahvaz Jundishapur University of Medical Sciences, Ahvaz, Iran; ^3^Students' Scientific Research Center (SSRC), Tehran University of Medical Sciences, Tehran, Iran

## Abstract

A 50-year-old man was admitted to our emergency department due to new episode of palpitation. He had history of angioplasty of right coronary artery (RCA) with drug eluting stent 2 years ago. His electrocardiogram revealed atrial fibrillation (AF). Intravenous amiodarone 150 mg during 10 minutes and then 1 mg/min infusion were started to achieve rate control and pharmacologic conversion to sinus rhythm. After 60 minutes of starting amiodarone infusion, he developed swelling of the skin around his mouth and eyes, and also mucosa of the mouth, eyes and tongue. To conclude, angioedema should be considered a rare side effect of amiodarone which is used broadly in cardiovascular field.

## 1. Introduction

Atrial fibrillation (AF) is the most familiar cardiac arrhythmia in clinical practice [1, 2]. Amiodarone, a class III long-acting antiarrhythmic drug, has been showed to be superior to other antiarrhythmic medications for the pharmacologic cardioversion of AF and maintenance of sinus rhythm especially in the setting of coronary artery disease and congestive heart failure [3–5].

## 2. Case Presentation

A 50-year-old man was admitted to our emergency department due to new episode of palpitation 3 hours ago. He had history of angioplasty of right coronary artery (RCA) with drug eluting stent 2 years ago. He had no complaint of chest pain, dyspnea, or palpitation until this morning when he woke up with palpitation. His drug history included aspirin 81 mg daily, metoprolol 50 mg daily, atorvastatin 20 mg daily, and isosorbide dinitrate 10 mg BID. On physical examination, blood pressure was 110/70, heart rate was 138 beats/min, respiratory rate was 14, and body temperature was 36.9°C. The remaining of his physical examination was not remarkable. His electrocardiogram revealed atrial fibrillation (AF) with the rate of about 130 beats/min with no ST segment or T wave changes.

Bed side transthoracic echocardiography demonstrated ejection fraction of 45%, inferior wall mild hypokinesia, mild diastolic dysfunction, and trivial mitral regurgitation.

Due to new atrial fibrillation and stable hemodynamic status, intravenous amiodarone 150 mg during 10 minutes and then 1 mg/min infusion were started to achieve rate control and pharmacologic conversion to sinus rhythm.

After 60 minutes of starting amiodarone infusion, he developed swelling of the skin around his mouth and eyes and also mucosa of the mouth, eyes, and tongue.

Moreover, he had complained of slight dyspnea; however, his hemodynamic state was stable and pulse oximetry showed 96% saturation with Oxygen in ambient air; so, tracheal intubation was not required.

Amiodarone infusion was immediately stopped and intravenous hydrocortisone 100 mg and chlorpheniramine 10 mg were introduced.

After 2 hours, his periocular and perioral swellings were made somehow better and he had no dyspnea or stridor; intravenous metoprolol was commenced for rate control.

His AF rhythm was not converted to sinus rhythm after 24 hours of admission; so, he underwent electrical synchronized cardioversion (150 J) that led to sinus rhythm.

The patient was discharged from hospital after four days in a good condition.

## 3. Discussion

Amiodarone has various side effects including ventricular arrhythmia, QT interval prolongation, abnormality in thyroid function, interstitial lung disease, liver enzymes elevation, and corneal deposits [6–11].

Long term administration of amiodarone is related with blue gray discoloration of the skin which is sensitive to light [12]. However, angioedema is extremely rare as a side effect of amiodarone, and there are few reports about this very uncommon reaction to amiodarone that almost always occurred in chronic use of amiodarone [13, 14]. To the authors' knowledge, this case is the first report of acutely onset amiodarone-induced angioedema.

As a conclusion, angioedema should be considered as a rare side effect of amiodarone which is used broadly in cardiovascular field.
